# The Surface Anatomy of the Heart and Aorta

**Published:** 1901-09-28

**Authors:** Seymour Taylor

**Affiliations:** Physician to the Hospital


### Hospital Clinics and Medical Progress.

### THE SURFACE ANATOMY OF THE HEART AND AORTA.

Being an abstract of a lecture delivered at the West London Hospital by Seymour Taylor, M.D., F.R.C.P., Physician to the Hospital.

The heart is not, as many would suppose, entirely on the left side of the body. In a mesial section, through the thorax from above downwards, the heart would be divided not quite equally—about seven-twelfths being on the left side of the section and five-twelfths on the right. The upper border of the heart reaches to the upper border of the third rib. The lower boundary may be indicated by a line drawn from the xiphoid cartilage to the apex-beat, which, as you know, is an inch lower than the nipple and half an inch internal to it. The right-hand boundary is one inch and a half from the mid-sternal line, the left-hand boundary is three and a half inches from the same line. In this parallelogram the heart is situated. The front part of the heart consists almost entirely of the right ventricle—a small part of the left ventricle coming to the front at the apex and at the base the tips of both auricles are seen—the right auricle being much more anterior than the left.

I may here remark that I am not in favour of the terms right and left ventricle. The right ventricle is anterior and the left ventricle is posterior, but if I could revise the nomenclature of the circulation I should be inclined to call the right ventricle the pulmonic ventricle, and the left, the systemic ventricle. Springing from the base of the heart we see the two trunk vessels, the aorta and the pulmonary artery, the former being behind the latter. The first inch or two of both vessels are contained in the pericardial sac. The cardiac valves are all of them close together, and they would be covered by a disc a little larger than a crown piece. The situation of these valves from above downwards is as follows:—the pulmonic, the highest, is at the left border of the sternum in the second space. The aortic valve is behind the sternum a little to the left of the middle line and at the level of the third rib. The mitral valve is in the third space almost below the pulmonic, but a little bit to the left of it, whilst the tricuspid valve is right in the middle line at the level of the fourth rib.

The aortic arch demands our attention for a few minutes on account of its important relations. Springing from the systemic ventricle beneath the sternum and one inch below the ridge which marks the junction of the manubrium with the gladiolus, it passes upwards and outwards to the right and is found to occupy the second right intercostal space immediately external to the right border of the sternum. The thrust of a knife into this space just external to the sternum therefore would be calculated to open the aorta and would be immediately fatal. The second part of the arch erroneously called "transverse" since it runs from before backwards to the third dorsal vertebra, runs beneath the manubrium, being midway between the sternal notch above and the aforesaid ridge where the manubrium joins the gladiolus. The third part of the arch lies upon the body of the third and fourth dorsal vertebra. The relations of these three arbitrary divisions are important in the diagnosis of aneurysm, but more especially in the diagnosis of the site of the aneurysm.

The important relations of the first portion are mainly those connected with the venous system. The superior cava is to the right, the pulmonary artery in front and to the left, whilst that division of the pulmonary artery which goes to the right lung is immediately behind it. It follows from this that an aneurysm of the first portion of the arch would be expected to impinge upon the pulmonary artery or its right branch; or again, as more frequently happens, it presses upon the superior cava and so causes bilateral or symmetrical œdema of the head and neck and both upper extremities. The more important relation of the second part of the arch are the trachea and œsophagus behind, and the vagus, phrenic and sympathetic nerve trunks in front. An aneurysm in this locality would be expected to cause tracheal tugging and brassy cough; but, more important still, affections of the recurrent laryngeal nerve, causing abductor palsy of the left vocal cord. This part of the arch also has running across it the left innominate vein, and an aneurysm springing from the convexity of this portion might be supposed to cause œdema only of the left side of the head and neck, and of the left arm. Inequalities of pulse might also arise by reason of the origins of the innominate, left carotid and left subclavian trunk vessels from this locality. The pressure signs of aneurysm of the third part of the arch are as a rule limited to obstruction of the œsophagus, (dysphagia), pressure upon the left bronchus and erosion of the dorsal vertebræ; but it must not be supposed for a moment that all these signs are revealed to us in the sequence that my demonstration would lead you to suspect. Aneurysms have a faculty of extending in different directions at different periods; hence pressure signs may vary from time to time in a manner which will cause some bewilderment when any attempt at diagnosis of their exact position is made.

The descending thoracic aorta lies deep in the thorax on the front of the vertebral column and passing downwards perforates the diaphragm at the twelfth dorsal vertebra. It is in this situation that aneurysm is most difficult of diagnosis, inasmuch as it would afford no signs which are conclusive and which are not found in common with malignant and other new growths of the posterior mediastinum.

I must devote a few words to the description of the œsophagus in the thorax before I bring this demonstration to a close. This muscular tube passes down the neck directly on the cervical vertebræ. It enters the thorax through the upper opening, runs behind the second part of the aortic arch, still behind the trachea; then passes internal to the third portion of the arch and passes down in front of the descending thoracic aorta and perforates the diaphragm at about the level of the ninth dorsal vertebra.

This muscular tube is very mobile and accommodative to hard morsels which may be quickly swallowed. In an obstruction of the œsophagus, the site of the referred pain is no necessary guide to the site of the stricture, but I would caution each of you to be extremely careful in passing an œsophageal tube, whether it be for diagnostic purposes or whether it be for the object of feeding the patient, since the muscular coats at best are very thin, and in the case of any malignant structure or any pressure on the gullet from aneurysm, the procedure may be fraught with the utmost peril to the patient. The seat of stricture is located by a tube being passed down to the obstruction; the distance being then measured from the incisor teeth. The distance from the incisor teeth to the cardiac orifice of the stomach is about 13 or 14 inches.

